# µRA—A New Compact Easy-to-Use Raman System for All Hydrogen Isotopologues

**DOI:** 10.3390/s22103952

**Published:** 2022-05-23

**Authors:** Florian Priester, Alexander Marsteller, Simon Niemes, Nancy Tuchscherer, Stefan Welte

**Affiliations:** Tritium Laboratory Karlsruhe, Institute for Astroparticle Physics, Karlsruhe Institute of Technology, 76131 Karlsruhe, Germany; alexander.marsteller@kit.edu (A.M.); simon.niemes@kit.edu (S.N.); nancy.tuchscherer@kit.edu (N.T.); stefan.welte@kit.edu (S.W.)

**Keywords:** Raman, spectroscopy, tritium, fiber optical system, Tritium Laboratory Karlsruhe

## Abstract

We have developed a new compact and cost-efficient Laser-Raman system for the simultaneous measurement of all six hydrogen isotopologues. The focus of this research was set on producing a tool that can be implemented in virtually any existing setup providing in situ process control and analytics. The “micro Raman (µRA)” system is completely fiber-coupled for an easy setup consisting of (i) a spectrometer/CCD unit, (ii) a 532 nm laser, and (iii) a commercial Raman head coupled with a newly developed, tritium-compatible all-metal sealed DN16CF flange/Raman window serving as the process interface. To simplify the operation, we developed our own software suite for instrument control, data acquisition, and data evaluation in real-time. We have given a detailed description of the system, showing the system’s capabilities in terms of the lower level of detection, and presented the results of a dedicated campaign using the accurate reference mixtures of all of the hydrogen isotopologues benchmarking µRA against two of the most sensitive Raman systems for tritium operation. Due to its modular nature, modifications that allow for the detection of various other gas species can be easily implemented.

## 1. Introduction

Having a precise knowledge of gas compositions during gas handling is a key factor for efficient process control. Dealing with all six hydrogen isotopologues can be a challenging task in regards to the available and compatible devices due to the issues that arise with the presence of radioactive tritium and material compatibility. Requirements, such as a very low leak rate (all-metal sealing) and non-invasive measurements, can be of interest for other applications, e.g., (i) high-purity gas handling, (ii) composition monitoring in industrial or research operations, and (iii) natural gas and oil processing. The system must be able to handle corrosive and explosive substances, both in gaseous and liquid forms. An established method is the use of mass spectrometry, which has the drawbacks of requiring a pressure of <10^−5^ mbar in order to be operational (which implies the use of turbomolecular pumps) and an inherent ambiguity due to isotopologues-mass signal overlap [1]. Tritium-compatible gas chromatography (GC) systems are, among other drawbacks, bulky, have a long analysis time, produce radioactive off-gases, and are virtually no longer commercially available [2,3,4]. Laser Raman spectroscopy is more suitable for the pressure regimens usually found in a typical tritium-handling system at TLK (50 mbar–900 mbar) [5,6,7]. The drawbacks of available systems are often related to either non-tritium compatibility or being a rather bulky and complex system.

Based on good experiences with the science-driven high-performance laser Raman system (LARA) [7], which is in use at the Tritium Laboratory Karlsruhe (TLK) for the 24/7 monitoring of the gas composition circulating inside the KATRIN experiment (40 g tritium per day, >95% purity) [8], and other tritium process infrastructure, we developed a more process-control oriented and straightforward device, called the “micro-Raman” (µRA) system (in the style of µGC). The focus of this development was the construction of a compact device that can be deployed within any existing gas-handling setup using standard interfaces (1/2″ VCR or DN16CF compatible) without the need for extensive modifications to existing glove boxes. By adjusting the measurement range of the Raman system (laser, spectrometer), this device can detect Raman scattered light in a 180° Raman setup in a wide range from approx. 200 nm up to 1000 nm making it suitable for more than “just” hydrogen isotopologues.

While there are numerous options for fully-integrated Raman systems commercially available (too many to name them all), they mostly lack modularity and unrestricted access to raw data and/or hardware control. Choosing the spectrometer and laser option from an even broader choice was a tradeoff and optimization between the availability of different configurations and combinations, functionality, and price.

This paper will introduce the setup, present the results regarding the performance of the system, and present the first results of the already deployed systems at the tritium infrastructure systems of TLK, thus focusing on hydrogen isotopologues. This includes the Tritium Transfer System (TTS) and the Isotopic Separation System (ISS) [9].

## 2. Materials and Methods

The µRA system relies on the Raman effect [10], where a laser excites a molecule which, in turn, emits light while it de-excites. This de-excitation produces light with a longer wavelength compared to the exciting laser light. By using the aforementioned 180° geometry, the same lens for focusing laser light and Raman scattered light was used, eliminating the need for precise alignment procedures for the excitation and collection side.

**Figure 1:**
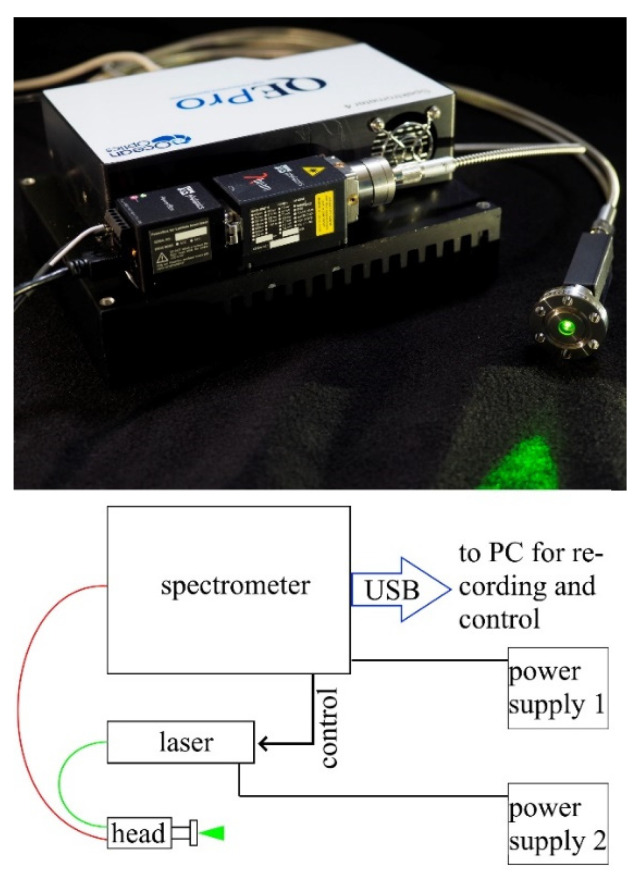
On top, the complete µRA system with laser, spectrometer, and Raman head with DN40CF flange. The heat sink measures 15 cm × 20 cm, which covers the whole system’s space needs. Bottom: Schematic of the connections between components. The green fiber guides the excitation laser to the specimen, the red fiber collects the Raman scattered light. All hardware control runs over a single USB connection, the laser is remote controlled via the spectrometer multi I/O port.

The µRA System (Figure 1) consists of three major parts: (1) a laser, (2) a 180° Raman head containing optical components and providing the interface to the gas, and (3) a spectrometer combined with a CCD sensor. All of the components are linked with optical fibers. The custom in-house manufactured glove box feed-throughs are not shown here.

The laser and spectrometer were mounted on a common heat sink for thermal management and easy handling. µRA used a 532 nm/0.2 W lambda-beam laser from rgb-Lasersystems. The spectrometer was a QEPro from the OceanInsight company in a custom configuration with an actively cooled 1024 pixel linear-CDD detector. The part in direct contact with tritiated gas, the Raman head/window combination, was developed in cooperation by TLK and the American company SpectraSolutions INC.

The device included all of the necessary optical elements (beam splitter, laser line filter, lenses, etc.) and the tritium-compatible interface (DN16CF) for coupling laser light into the gas volume and Raman light out. The Raman head/window were the only components placed inside the glove box. The head had a bandpass filter on the excitation train matching the laser wavelength, while the actual DN16CF window housed the collimation lens for laser focusing and Raman light collection. The wavelength could be chosen to match an application (we used a 12 mm focal length for the setup described). The head/window could be operated up to a laser power of 2 W cw.

With the window being the element enclosing the pure tritium up to ≈900 mbar, high requirements with regard to leak tightness and material selection were applied. Laser light and Raman signal were transmitted by two independent fibers through the glove box barrier. This allowed for the maintenance of most of the components to be very easy (no contamination) and saved valuable space inside the box. Laser light was transmitted by a single 105 µm core diameter fiber (Fiberguide AFS105/125/250Y), while for the Raman light collection, we used a 6-around-1 configuration (seven 100 µm fibers, step index, multi-mode in a round to linear configuration) custom made by OceanInsight. Both assemblies used fused silica as the fiber material. The spectrometer offered a multitude of settings to manipulate a spectrum where only a small subset was used for all of the presented data (see “Determining the systems’ characteristic and performance” for details). For all of the measurements at TLK, we developed a custom Python-based analysis software tailored to the needs of hydrogen-isotopologue spectroscopy and day-to-day operation. We aimed for a stable and easy-to-use platform; a standard measurement could be started with just two clicks. All of the important parameters were stored in human-readable/editable configuration files containing all of the settings for the recording of successful operation/data and data evaluation. The software provided a close-to-real time display of:

- the spectrum (with options for background subtraction),
- the relative concentration (numerical) and trend view of all six hydrogen isotopologues
- the relative atomic concentration (H, D, T),

and additionally

- saving of raw spectral data (always-on),
- export of trend values of all six hydrogen isotopologues.

All functionality is available for offline data for easy re-evaluation of previously recorded data.

The calculation of all of the relative concentrations was based on a configuration file containing information about the line position, integration width (ROI), region for background subtraction, and correction factors. For the latter, we used a correction for the relative line strength of all isotopologues [5,11] and calibration values from our in-house calibration using an NIST-certified Raman standard [12].

The flexibility comes from user-definable gas species such as N_2_, CQ_4_ (Q = H, D, T), or H_2_O being added to the list. Once prepared, the µRA could evaluate the signals of such species as well. If there is a need to detect Raman scattered light outside the range of the current setup, using another excitation wavelength and/or adjusting the spectrometer’s grid can be facilitated easily.

**Figure 2:**
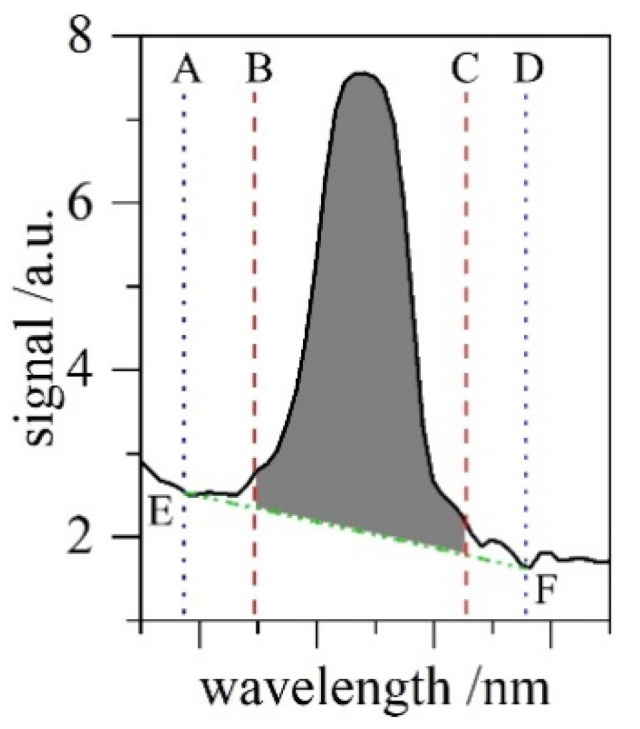
Visualisation of the peak area determination. The software determines the flat grey area of each pre-defined peak region. Refer to text for further details.

The calculation of the relative concentrations worked as follows, referring to Figure 2:

The configuration file contains information about the start-/endregion of a peak (B–C, red) and a definition for the region defining the signal background at a given position (A–D, blue). The software interpolated a straight line between the intersection of A and D with the signal (E–F, green), assuming a linear background for that wavelength interval. Everything between B–C and above E–F was numerically integrated (flat grey). This was performed for each pre-defined region in every single spectrum, giving the relative composition of all of the isotopologues.

There is an option to actively ignore certain species during the concentration calculation in case the user is sure that there is, e.g., no tritium (T_2_/DT/HT) in a sample, which will improve the accuracy of the other isotopologues since a possible noise at a defined peak region will be ignored. A report in a .pdf file format, based on a freely configurable .html template, can be generated from within the software.

## 3. Results and Discussion

In Section 3.1, we present the results of the initial test series with only H_2_ and D_2_ (and mixtures thereof), while Section 3.2 will give the results of the measurements at two of the TLK’s infrastructure systems, the TTS and the ISS.

### 3.1. µRA Characteristics and Performance

Prior to the first measurements with tritium, the complete system was characterized by inactive gases, i.e., H_2_, D_2_, and HD. This was undertaken to assess the systems’ overall performance and response. During these measurements carried out at the HyDe-Loop [13], the optimized parameters for data acquisition and the systems’ capabilities regarding the lower level of detection were determined. For those tests, our own software was not yet ready, so we used OceanInsight’s own software suite, which—in our case—only recorded raw data. The background subtraction and peak identification were conducted offline afterward, without relative line-strength or spectrum correction applied. The following settings were found most suited for standard operation:

The µRA uses (i) three-fold averaging (three spectrums averaged for stability and noise suppression), (ii) electric dark-current offset compensation, and (iii) a boxcar filter with the width of three channels. A standard acquisition time was 20 s/spectrum. All of these operations were performed on the spectrometer’s own CPU; thus, only a single dataset was transferred each minute to a connected computer. These settings apply to all of the data presented in this paper unless otherwise stated.

**Figure 3:**
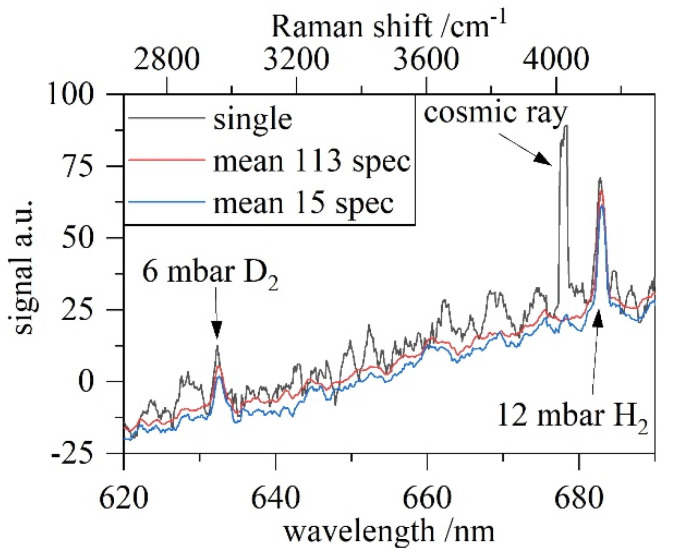
Part of the “pump down test” series of measurements with a total pressure of ≈19 mbar, with ≈6 mbar ± 0.5 mbar D_2_ and 12 mbar ± 0.5 mbar H_2_.

Amongst other mixtures, we used a non-equilibrated binary mixture of 75 mbar absolute pressure containing 2/3 H_2_ and 1/3 D_2_. By pumping down the well-mixed gas inventory, we were able to determine the performance of the system at different (partial-) pressures. Figure 3 shows a typical result obtained during the “pump down test” series with the fluorescence background removed. A fluorescence background is present in all Raman systems and can change in shape and intensity depending on the mounting situation. The single measurement at 19 mbar already shows sufficient signal strength where the relative content of H_2_/D_2_ can be extracted. Smoothing the spectrum by calculating the mean value of 15 spectrums (=15 min measurement time) reduces the noise floor already significantly. The further improvement for 113 averaged spectrums is neglectable. It is noteworthy that unavoidable cosmic-ray events might fall into a pre-defined ROI, mimicking a Q_2_ signal. This influence can be reduced by simple averaging over short time scales.

**Figure 4:**
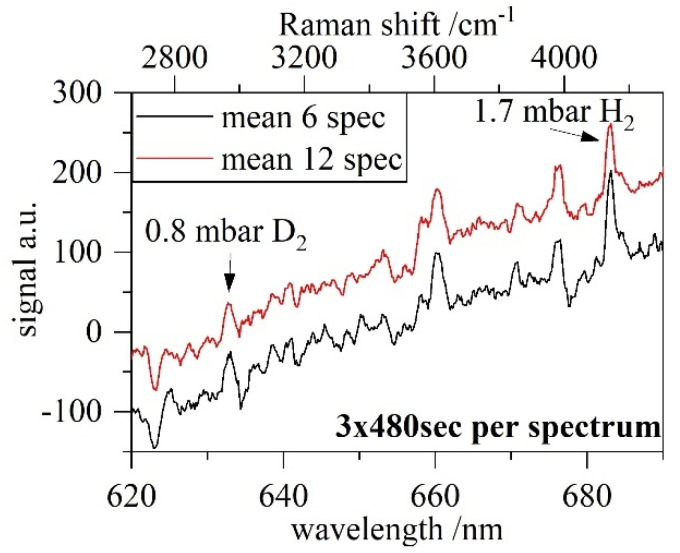
Lowest achieved pressure of ≈2.5 mbar, resulting in ≈ 0.8 mbar ± 0.5 mbar D_2_ and 1.7 mbar ± 0.5 mbar H_2_ during the “pump down test” series. Note the different acquisition time.

When pushing µRA to the limit, we needed to adjust the acquisition time to 8 min/spectrum with all of the other settings unchanged. Figure 4 shows the lowest compositional measurement by the µRA system achieved so far of ≈2.5 mbar ± 0.5 mbar absolute, resulting in a D_2_ partial pressure in the range of 0.5–1 mbar. After averaging ≈2.5 h of measurement time (mean 3 × 6 spectrum) a signal can be extracted. While taking some time, this setting is still useful if ultimate precision and LOD are required for static samples. Coming back to standard settings and common pressures of 100–900 mbar, reliable measurements limited by statistical uncertainty can be easily obtained within 10–15 min, with the first calculated results available within 3 min.

### 3.2. µRA at TLK Infrastructure Systems

While deploying the µRA systems at TLK’s infrastructure systems—the TTS and ISS [9]—our own software was available for data taking. It uses the exact same settings as for all previous measurements but automates all of the analysis steps. In addition, it also uses corrections for (i) relative line intensity [11] and (ii) spectral sensitivity [12] for displaying and calculating the relative concentrations but still saves the unchanged (raw) spectral data.

#### 3.2.1. TTS

For cross-checking, the µRA was mounted to a sampling port of the TTS close to the LARA [7] system, which has been used for some years for tritium accountancy. High-precision reference gas samples were provided by the TriHyDe facility [5]. Each gas sample was cross-checked with multiple independent methods and devices for its composition, including the analytical tools of TriHyDe (a 2nd LARA system and a new device utilizing the speed of sound for compositional analysis (BGA) [6]).

Table 1 summarizes the prepared and analyzed five samples in total, ranging from mixtures with no T_2_ to samples up to 50% T_2_. All of the samples were measured with µRA at around the same pressure of ≈197 ± 5 mbar to be comparable. The lines “at. H”, “at. D”, and “at. T” in Table 1 give the initial relative atomic composition, which was subsequently equilibrated [6] in TriHyDe (except #138). Table 1 summarizes all five runs (#134–#138), giving the relative concentration measured by the µRA system, both LARA systems (at TTS and at TriHyDe), as well as the expectation from theoretical calculations [14]. The speed of sound (BGA) measurements all agreed within <0.5% with the initial mixture composition and are therefore not listed separately. Detailed information on how the BGA method works can be found in [6].

**Figure 5:**
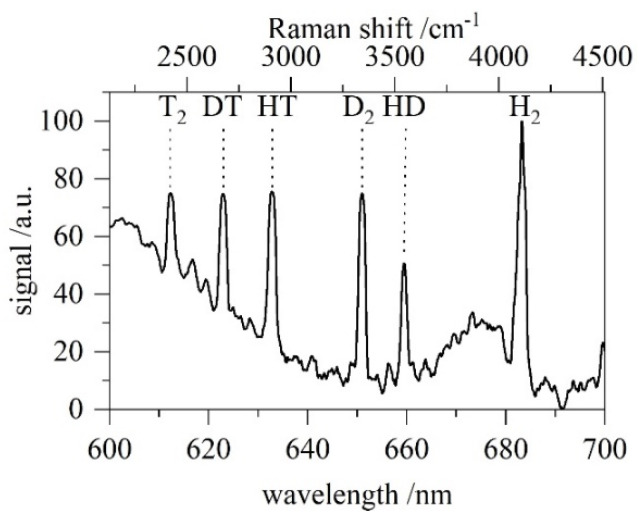
Spectrum obtained during the calibration campaign. For convenience, all five measurements are displayed in one diagram showing nicely the well-separated signals of the isotopologues.

**Figure 6:**
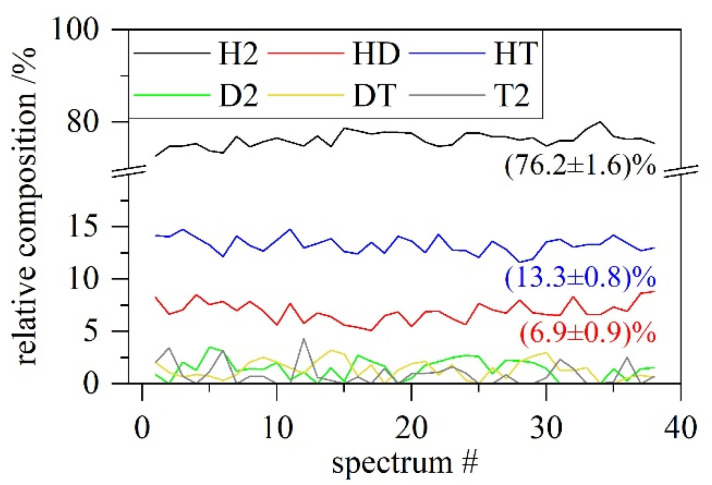
Time evolution of all isotopologues during gas sample #134. This covers 38 min (1 spectrum per minute) of measurements. Mean value and error (1σ standard deviation) are given for the most prominent species.

Table 1 shows the good agreement between the theory and µRA results with a tendency to slightly underestimate heavy isotopologues and overestimate lighter ones. This can be based on possible gas-wall interactions, such as isotope exchange during sample transfers. Further possible causes are non-linearities in the detector (which are not accounted for) or the extrapolation of the NIST standard towards longer wavelengths, i.e., H_2_. This can be easily fixed with a correction factor in case future measurements verify that tendency. Even without further correction, the deviation is <5% for extreme cases and <3% for the majority of all values. Using the same settings and sample pressure across all five test gases, the integrated spectrum is given in a single diagram (Figure 5). It contains all six hydrogen isotopologues, showing the good separation of each peak and the unambiguous signal superposed with a fluorescence background. Figure 6 displays the time evolution of each isotopologue of a single mixture. As an example, the measurements from the first gas sample (#134, Table 1) were taken.

#### 3.2.2. ISS

Two more µRA systems were installed at the ISS, enabling the in situ monitoring of gas composition in the product vessels during the separation while withdrawing the different product gas streams. Only the compact size and flexibility through fiber coupling allowed it to be possible to install the systems on top of each product vessel monitoring the gas feed to it or a static composition after the separation process. As a result, µRA additionally eliminates time-consuming sample transfer via double-walled lines to the analytical system of the TTS for composition check.

## 4. Conclusions and Outlook

We have successfully designed and operated the new compact µRA instrument for the in situ measurement of all six hydrogen isotopologues. Benchmarking the µRA results against proven high-end Raman systems shows that—even with a significantly lower laser power—the results are comparable for daily process control work to the results of the more sophisticated Raman system used for many years at TLK [7] and for commonly used gas pressures in tritium processing systems. Due to µRA’s small size and easy usability, it was possible for the first time in TLK’s 25-year long history to have close-to real-time monitoring of the isotope separation process in the ISS.

The perfectly tailored software environment allowed for straightforward day-to-day operation while leaving the option to further expand the list of detectable gases with ease.

In contrast to commercially available compact all-in-one Raman solutions (with integrated laser, spectrometer, and data evaluation), we achieved full access to all of the vital data and hardware control. This opens up the possibility of replacing, e.g., the laser with more powerful options up to 2 W cw for even lower levels of detection or shorter acquisition times. In conjunction with the newly developed all-metal sealed Raman window with integrated Raman head, which is available for the first time, µRA-enabled valuable Raman measurements in formerly non-accessible, existing applications. The in-house development of our own data control and evaluation software provides full control over all steps taken, not relying on a proprietary software environment. With software development in our hands, it is even possible to integrate the µRA systems in typical process control systems for remote and automated operation.

We intend to install further µRA systems at TLK, e.g., replacing the aged and decommissioned gas chromatography (GC) system at the CAPER facility [9] with a µRA system in combination with a mass spectrometer. We will continuously monitor the long-term stability of the measurements and the components, especially the ones in direct contact with tritium, to ensure the high long-term usability of the whole system. With planned upgrades and modifications, we intend to further enhance and extend the capabilities of the system to allow a wide range of applications, including non-tritium-related use cases.

## Figures and Tables

**Table 1:** Summary of all gas mixtures (#134–#138) used for characterization and comparison. Each sample was analyzed with the µRA device, a “conventional” LARA system [7] at TriHyDe [5,6] and TTS [9]. In addition, theoretical expectation values are given for comparison. The entries at. H /at. D/at. T gives the initial mixture, e.g., 50% at. T and 50% at. H means an initial 1:1 mixture of H_2_ and T_2_. #138 was not equilibrated.

Species	#134				#135				#136				#137				#138			
%	µRA	TriHyDe	TTS	theo.	µRA	TriHyDe	TTS	theo.	µRA	TriHyDe	TTS	theo.	µRA	TriHyDe	TTS	theo.	µRA	TriHyDe	TTS	theo.
H_2_	76.2	71.2	68.7	72.9	29.8	27.3	25.6	27.7	14.3	12.5	11.4	12.5	0.0	0.3	0.1	0.0	52.5	48.4	43.8	26.3
D_2_	1.4	0.7	0.3	0.0	1.2	0.2	0.1	0.0	12.3	11.2	12.1	11.1	28.7	25.9	25.2	25.3	44.8	50.0	54.3	26.3
HD	6.9	8.6	8.4	8.2	0.2	0.3	0.2	0.0	22.1	22.5	20.8	21.3	0.3	0.6	0.5	0.0	0.8	0.8	0.8	47.5
T_2_	0.9	1.8	3.8	1.4	23.7	25.7	28.0	27.7	11.8	11.3	11.8	11.9	23.0	23.7	25.1	25.3	1.2	0.3	0.2	0.0
HT	13.3	16.4	16.9	16.0	44.3	46.2	45.5	44.5	19.3	20.7	19.8	19.6	0.2	0.3	0.5	0.0	0.1	0.3	0.1	0.0
DT	1.3	1.3	1.5	1.2	0.8	0.4	0.5	0.0	20.2	21.8	24.0	22.5	47.7	48.8	48.2	49.4	0.6	0.2	0.4	0.0
at. H	86.3	83.7	81.4	85.0	52.0	50.5	48.4	50.0	35.0	34.1	31.7	33.3	0.3	0.8	0.6	0.0	53.0	49.0	44.2	50.0
at. D	5.5	5.6	5.3	5.0	1.7	0.5	0.4	0.0	33.4	33.4	34.5	33.3	52.7	50.6	49.6	50.0	45.4	50.5	54.9	50.0
at. T	8.2	10.6	13.0	10.0	46.3	49.0	50.9	50.0	31.6	32.6	33.7	33.3	47.0	48.3	49.5	50.0	1.6	0.5	0.5	0.0

## Data Availability

The data presented in this study are available on request from the corresponding author. The data are not publicly available due to non-proliferation regulations.
